# Supplementary material for: Modeling glioblastoma heterogeneity as a dynamic network of cell states
Source: Mol Syst Biol. 2021 Sep 16;17(9):e10105. doi: 10.15252/msb.202010105 (PMC8444284; doi:10.15252/msb.202010105)
Supplement: Supplementary file 6 — Source Data for Figure 5 [file MSB-17-e10105-s004.zip › Figure5A_sourcedata/GSEA_3017/nosigdb_stateC.GseaPreranked.1622795002785/OLSEN_SCPLIKE.html]

Details for gene set OLSEN\_SCPLIKE[GSEA]

|  || Dataset | state13017 |
| Phenotype | NoPhenotypeAvailable |
| Upregulated in class | na\_neg |
| GeneSet | OLSEN\_SCPLIKE |
| Enrichment Score (ES) | -0.59206444 |
| Normalized Enrichment Score (NES) | -3.050595 |
| Nominal p-value | 0.0 |
| FDR q-value | 0.0 |
| FWER p-Value | 0.0 |
Table: GSEA Results Summary

  

Fig 1: Enrichment plot: OLSEN\_SCPLIKE      
 Profile of the Running ES Score & Positions of GeneSet Members on the Rank Ordered List

  

| PROBE | GENE SYMBOL | GENE\_TITLE | RANK IN GENE LIST | RANK METRIC SCORE | RUNNING ES | CORE ENRICHMENT || 1 | IGFBP7 |  |  | 225 | -0.259 | -0.3829 | No |
| 2 | AHR |  |  | 324 | -0.286 | -0.5385 | No |
| 3 | COL4A2 |  |  | 353 | -0.300 | -0.5689 | Yes |
| 4 | S100A11 |  |  | 361 | -0.301 | -0.5619 | Yes |
| 5 | PMEPA1 |  |  | 375 | -0.308 | -0.5651 | Yes |
| 6 | IGFBP5 |  |  | 384 | -0.311 | -0.5592 | Yes |
| 7 | PLP1 |  |  | 386 | -0.312 | -0.5408 | Yes |
| 8 | TIMP1 |  |  | 399 | -0.320 | -0.5414 | Yes |
| 9 | S100A6 |  |  | 423 | -0.336 | -0.5605 | Yes |
| 10 | PMP2 |  |  | 424 | -0.337 | -0.5388 | Yes |
| 11 | RHOC |  |  | 455 | -0.359 | -0.5689 | Yes |
| 12 | EDNRB |  |  | 462 | -0.367 | -0.5559 | Yes |
| 13 | B2M |  |  | 468 | -0.371 | -0.5408 | Yes |
| 14 | LGALS3 |  |  | 471 | -0.374 | -0.5202 | Yes |
| 15 | IFITM2 |  |  | 483 | -0.388 | -0.5147 | Yes |
| 16 | AP1S2 |  |  | 489 | -0.395 | -0.4981 | Yes |
| 17 | CD9 |  |  | 515 | -0.443 | -0.5138 | Yes |
| 18 | CST3 |  |  | 520 | -0.452 | -0.4917 | Yes |
| 19 | IFITM3 |  |  | 533 | -0.477 | -0.4822 | Yes |
| 20 | ITGA6 |  |  | 535 | -0.479 | -0.4531 | Yes |
| 21 | CRYAB |  |  | 536 | -0.485 | -0.4218 | Yes |
| 22 | VMP1 |  |  | 542 | -0.502 | -0.3982 | Yes |
| 23 | COL4A1 |  |  | 545 | -0.507 | -0.3690 | Yes |
| 24 | TIMP3 |  |  | 553 | -0.530 | -0.3472 | Yes |
| 25 | PLAT |  |  | 556 | -0.538 | -0.3160 | Yes |
| 26 | S100B |  |  | 561 | -0.561 | -0.2869 | Yes |
| 27 | ANXA2 |  |  | 563 | -0.567 | -0.2521 | Yes |
| 28 | MT2A |  |  | 566 | -0.575 | -0.2185 | Yes |
| 29 | SPARC |  |  | 567 | -0.575 | -0.1814 | Yes |
| 30 | S100A16 |  |  | 569 | -0.582 | -0.1455 | Yes |
| 31 | VIM |  |  | 578 | -0.674 | -0.1162 | Yes |
| 32 | NEAT1 |  |  | 579 | -0.683 | -0.0720 | Yes |
| 33 | S100A10 |  |  | 582 | -0.691 | -0.0309 | Yes |
| 34 | PMP22 |  |  | 590 | -0.837 | 0.0107 | Yes |
Table: GSEA details [plain text format]

  

Fig 2: OLSEN\_SCPLIKE: Random ES distribution      
 Gene set null distribution of ES for **OLSEN\_SCPLIKE**
